# Measurement of Cyanine Dye Photobleaching in Photosensitizer Cyanine Dye Conjugates Could Help in Optimizing Light Dosimetry for Improved Photodynamic Therapy of Cancer

**DOI:** 10.3390/molecules23081842

**Published:** 2018-07-24

**Authors:** Nadine S. James, Ravindra R. Cheruku, Joseph R. Missert, Ulas Sunar, Ravindra K. Pandey

**Affiliations:** 1 PDT Center, Cell Stress Biology, Roswell Park Cancer Institute, Buffalo, NY 14263, USA; nadine.james@roswellpark.org (N.S.J.); ravindra.cheruku@roswellpark.org (R.R.C.); joseph.missert@roswellpark.org (J.R.M.); ulas.sunar@roswellpark.org (U.S.); 2 Department of Biomedical Engineering, Wright State University, Dayton, OH 45435, USA

**Keywords:** Photodynamic therapy, photobleaching, photosensitizers, fluorescence imaging

## Abstract

Photodynamic therapy (PDT) of cancer is dependent on three primary components: photosensitizer (PS), light and oxygen. Because these components are interdependent and vary during the dynamic process of PDT, assessing PDT efficacy may not be trivial. Therefore, it has become necessary to develop pre-treatment planning, on-line monitoring and dosimetry strategies during PDT, which become more critical for two or more chromophore systems, for example, PS-CD (Photosensitizer-Cyanine dye) conjugates developed in our laboratory for fluorescence-imaging and PDT of cancer. In this study, we observed a significant impact of variable light dosimetry; (i) high light fluence and fluence rate (light dose: 135 J/cm^2^, fluence rate: 75 mW/cm^2^) and (ii) low light fluence and fluence rate (128 J/cm^2^ and 14 mW/cm^2^ and 128 J/cm^2^ and 7 mW/cm^2^) in photobleaching of the individual chromophores of PS-CD conjugates and their long-term tumor response. The fluorescence at the near-infrared (NIR) region of the PS-NIR fluorophore conjugate was assessed intermittently via fluorescence imaging. The loss of fluorescence, photobleaching, caused by singlet oxygen from the PS was mapped continuously during PDT. The tumor responses (BALB/c mice bearing Colon26 tumors) were assessed after PDT by measuring tumor sizes daily. Our results showed distinctive photobleaching kinetics rates between the PS and CD. Interestingly, compared to higher light fluence, the tumors exposed at low light fluence showed reduced photobleaching and enhanced long-term PDT efficacy. The presence of NIR fluorophore in PS-CD conjugates provides an opportunity of fluorescence imaging and monitoring the photobleaching rate of the CD moiety for large and deeply seated tumors and assessing PDT tumor response in real-time.

## 1. Introduction

PDT was initially developed for the local destruction of solid tumors [1,2] and is currently being used worldwide in the treatment of several tumors including skin basal cell carcinoma (BCC) [3], lung [4,5,6], esophagus [7,8,9,10,11], bladder, head and neck [4,6,12], brain [13,14,15,16,17], ocular melanoma, ovarian, prostate [8,9,10,11,12,13,14,15,16,17,18,19,20], renal cell, cervix, pancreas and bone [21]. It is also being used for a plethora of additional indications such as dysplasia, papillomas, rheumatoid arthritis, age related macular degeneration, actinic keratosis, cosmesis, psoriasis, endometrial ablation, localized infection (bacterial and fungal) and prophylaxis of arterial restenosis. Considering that the use of PDT has been approved for many diseases, it is still not being practiced in mainstream oncology. In part, this is because the therapy using the light dosimetry based on measured or calculated physical values is not yet optimized.

PDT is known to be dependent on three primary components: Photosensitizer (PS), light and oxygen in order to achieve an effective treatment [1]. Therefore, it is necessary to establish an understanding of the basic physical and biophysical interactions of these three essential components to maximize PDT output. Over the past two decades, much work has been done to optimize these components but their dynamic nature and interdependency lead to complexity. Therefore, a better understanding of PDT dosimetry is important so it will be a reliable tool for controlled enhancement of the therapeutic outcome.

Currently most of the photosensitizers (PS) used in PDT elicit significant damage to cancer cells through singlet oxygen mediated pathways [1]. The standard approach in clinical PDT involves administering a specified amount of photosensitizer (per body weight), wait for a specified time before applying a fixed amount of light {light fluence (J/cm^2^)} and fluence rate {total energy delivered within a specific drug-light time interval (mW/cm^2^)} [22] to treat each patient. However, this approach sometimes leads to incomplete or unpredictable responses in patient groups, which may be due to differences in individual physiological factors [22,23]. These heterogeneous factors include local tissue optical properties, tumor oxygenation and accumulated photosensitizer dose, which can be very different for each patient and tumor. These factors also may change during the PDT treatment. For example, photobleaching of the PS may reduce singlet oxygen production which may cause ground-state oxygen to be depleted if the reperfusion capacity of the tissue is exceeded by the immediate photochemical reaction [24]. Therefore, it has become necessary to develop pretreatment planning, on-line monitoring and dosimetry strategies during PDT [1].

Optimization of clinical dosimetry methods can follow one of three paths as described previously (see for example McIlroy et al. [24], Wilson et al. [25] and Zhu [26]) and can be classified as direct [26], explicit or implicit dosimetry [22,25]. Direct dosimetry involves the measurement of singlet oxygen itself, either through emission of its phosphorescence or through singlet oxygen sensitive chromophores [26]. Explicit dosimetry employs techniques and instrumentation to measure the three essential components of photodynamic therapy (light, photosensitizer (PS) and oxygen) individually and independently in the tissue [22,25]. A predictive model of the photobiological effect of these three components is required to combine the measurements into a dose metric [22,23,25]. Significant progress has been made in regard to the application of explicit dosimetry but there are still limitations [25]. Implicit dosimetry seeks to avoid measuring the light, PS and oxygen independently by eliciting the use of a single parameter that incorporates two or more of the essential components into a single metric in order to predict the biological damage [1,22,25,27]. This is chiefly accomplished by monitoring the PS photobleaching during irradiation by utilizing the fluorescence properties of the PS [22,25].

We are engaged in the exploration of PDT dosimetry strategies by employing the implicit dosimetry approach. In our strategy, we aim to investigate the utility of the photosensitizer-near infrared fluorophores conjugate (PS-NIRF) as fluorescence probes and markers for PDT light dosimetry. We hypothesize that the photobleaching characteristics of the fluorophores when subjected to variable light fluence and fluence rates in treating tumors with variable vascularity will help to optimize the light dosimetry and PDT response.

If singlet oxygen is the main cytoxic agent and the main cause of photobleaching, monitoring the photobleaching could provide a quantifiable measure of the singlet oxygen production. Therefore, we monitored the photobleaching of the photosensitizer and the fluorophore of several bifunctional photosensitizer-fluorophore conjugates using real time in vivo fluorescence imaging. In our initial attempt, the fluorescence of the NIR fluorophore portion of the conjugate was measured intermittently throughout the PDT treatment. First, in vitro photobleaching experiments were performed as a model of the in vivo systems to see if they could predict the in vivo response. Then, we imaged mice before and after PDT treatment at three different light doses, after exciting the PS or the NIRF portion of the conjugate individually and investigated potential direct correlation between photobleaching and PDT efficacy.

## 2. Results and Discussion

The degradation of the conjugates was observed via UV-Vis spectroscopy in vitro. Although these solutions of conjugates were made up as 5 μM solutions in 17% Bovine Calf Serum (BCS) it was understood that the intent was not to reproduce exact solvent conditions used clinically but to show whether drug aggregation or binding with serum affected the photobleaching of the chromophores. However, in our in vivo photobleaching experiments the degradation of the NIR fluorophore (CD) portion of the PS-CD conjugates was observed. In later experiments, we observed the degradation of both PS and CD via fluorescence quenching.

### 2.1. Mechanism of Photo-Induced Bleaching

**Figure 1:**
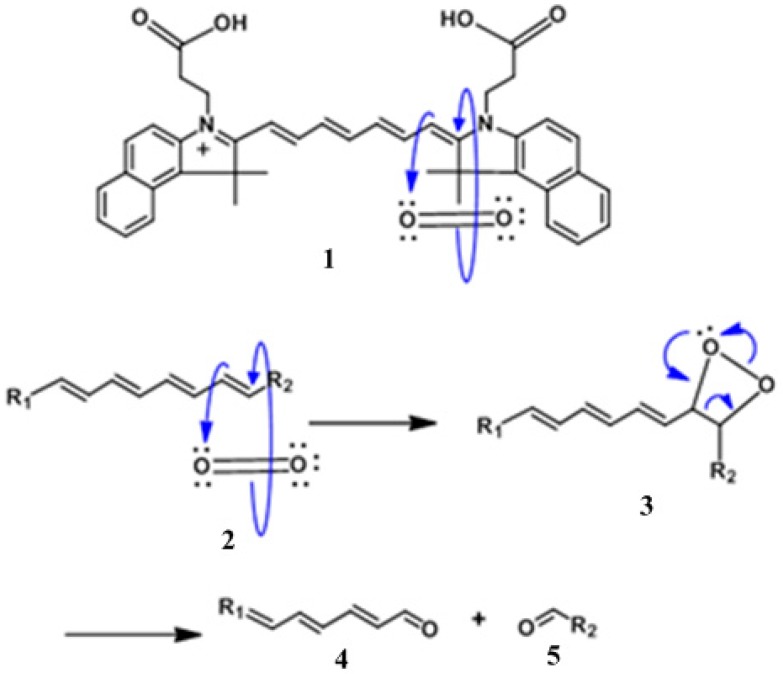
Mechanism of the photobleaching of Cypate by singlet oxygen. This illustrates the photobleaching that occurs in vitro and in vivo following absorbance of light in the near-infrared (NIR) region of the spectrum.

Using the NIR CD as a guide, Figure 1, illustrates the mechanism of photo-degradation caused by the contribution of molecular oxygen and light. Interaction of singlet oxygen with the chromophores constitutes the major pathway of photodecomposition. When the PS (HPPH) portion of the PS-NIR fluorophore (CD) conjugate absorbs light it undergoes intersystem crossing from an excited singlet state to an excited triplet state where it interacts with endogenous ground state triplet oxygen to generate the destructive singlet oxygen species. In the illustration below, the singlet oxygen generated subsequently attacks the polymethine chain of cypate resulting in fragmentation of the CD moiety as shown in Figure 1.

The photoproduct obtained after photo-induced bleaching is due to oxidation of the C′-C2 or C7′-C2″ bond on the polymethine chain. Singlet oxygen is directed towards the polymethine chain at the C’-C2 or C7′-C2″ bond due the electro-positivity of the 2 and 2″ carbon versus the electron rich position 1′ or 7′ of the cationic chromophore [28]. This degradation generates the corresponding carbonyl photoproducts [28].

### 2.2. In Vitro Photobleaching of HPPH Cyanine Dye and CD Conjugates

**Figure 2:**
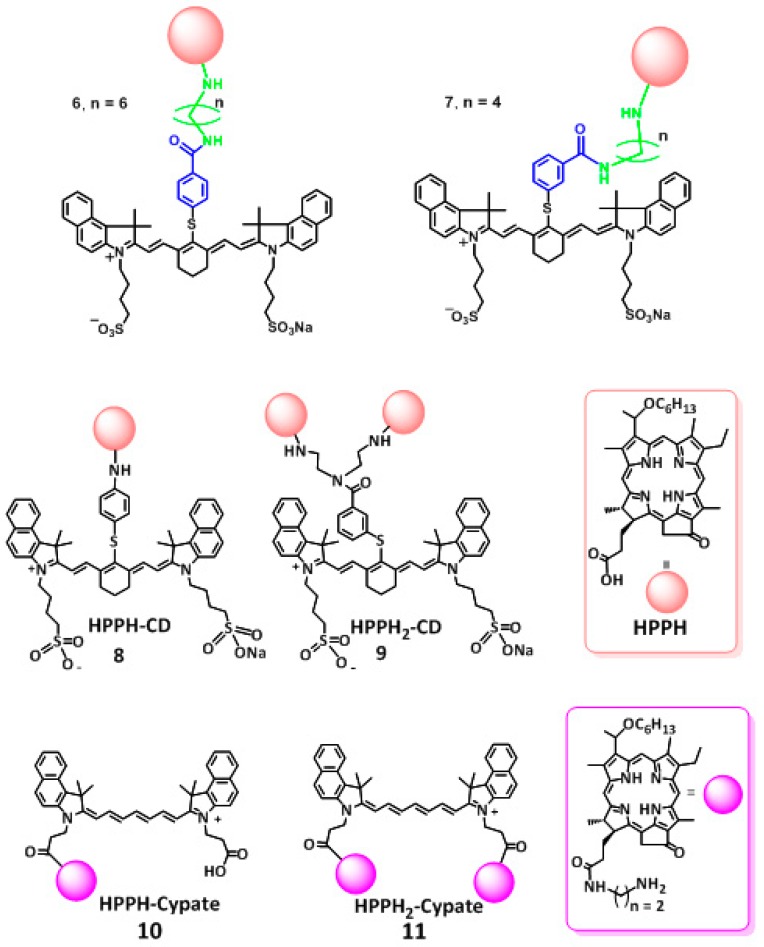
Structures of the conjugates **6**–**11** used for the study.

**Figure 3:**
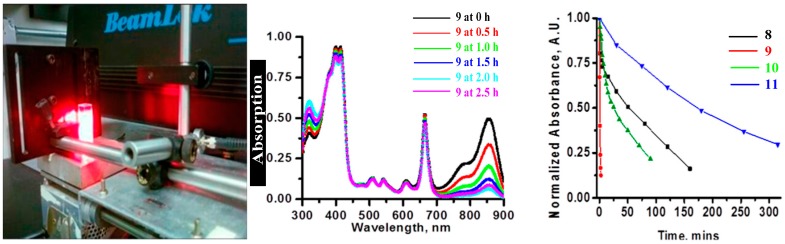
In vitro photobleaching rates of the conjugates at 5 μM solutions in 17% bovine calf serum (BCS) in phosphate buffer saline (PBS). HPPH-CD (**8**), HPPH_2_-CD (**9**), HPPH-Cypate (**10**) and HPPH_2_-Cypate (**11**) were in the order of **9** > **10** > **8** > **11**. The rate of photobleaching of the chromophores was determined on the basis of reduced absorption at 661 nm (HPPH) and near 830 nm cyanine dye (CD) after irradiating with light (135 J/cm^2^, 75 mW/cm^2^) at 661 nm (in vitro absorption wavelength).

The conjugates: HPPH-CD (**8**), HPPH_2_-CD (**9**), HPPH-Cypate (**10**) and HPPH_2_-Cypate (**11**) shown in Figure 2 prepared by following our own methodology [29,30] were formulated in 17% Bovine Calf Serum/PBS and were used at equimolar concentrations (5 μM). The absorption spectra were measured in 1 cm-quartz cuvettes following irradiation at 661 nm (light dose: 135 J/cm^2^, 75 mW/cm)^2^ at various time points until there was nearly complete degradation of the NIRF portion of the conjugate (Figure 3).

### 2.3. In Vivo Photo-Induced Bleaching Kinetics

**Figure 4:**
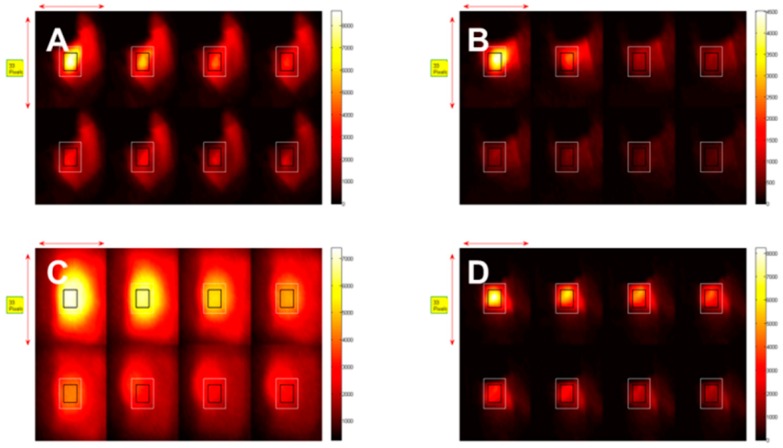
In vivo photobleaching of the CD in (**A**) HPPH-CD (**8**), (**B**) HPPH_2_-CD (**9**), (**C**) HPPH-Cypate (**10**) and (**D**) HPPH_2_-Cypate (**11**) conjugates in Colon26 tumors implanted in BALB/c mice. All conjugates were irradiated at 665 nm, after 24 h post-injection and treated at a fluence and fluence rate of 135 J/cm^2^ and 75 mW/cm^2^. Concurrently fluorescence images were taken (wavelength detection > 830 nm) at various treatment times up to 30 min, the total treatment time.

**Figure 5:**
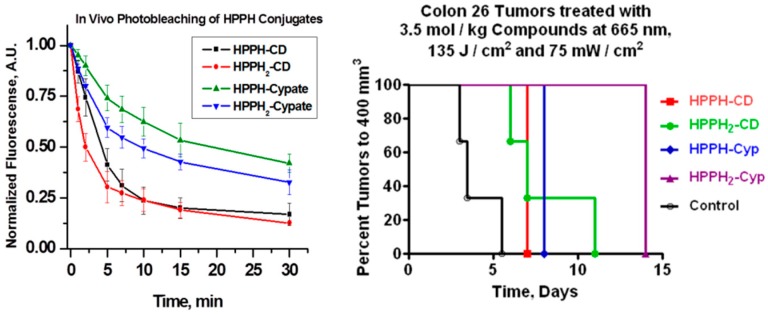
In vivo photobleaching of the conjugates: HPPH-CD **8**, HPPH_2_-CD **9**, HPPH-Cypate **10** and HPPH_2_-Cypate **11** occurred in the order of **9** > **8** > **11** > **10**. There were no photodynamic therapy (PDT) cures upon assessment of the tumor response (the tumors were irradiated with light (Figure 4) at 24 h post-injection). The tumor growth was measured daily, when the tumors reached to a volume of >400 mm^3^, the mice were euthanized following the approved IACUC protocol. Control: The tumored mice were intravenously (i.v.) injected with the PS but the tumors were not exposed to light.

BALB/c mice (3/group) were inoculated with compounds **8**, **9**, **10** and **11** and monitored during PDT for 30 min at various time intervals. The tumors were irradiated at a wavelength 665 nm using a total light dose of 135 J/cm^2^ and fluence rate of 75 mW/ cm^2^, respectively. Concurrently, the fluorescence kinetics were monitored by illuminating at 785 nm and measuring the fluorescence with an 830 long pass filter. Figure 3 shows images of the photo-induced bleaching kinetics of the four conjugates **8**, **9**, **10** and **11** at different time points during treatment. The mice injected with HPPH-Cypate (**10**) were irradiated at 665 nm. PDT efficacy of HPPH-CD (**8**), HPPH2CD (**9**), HPPH-Cypate (**10**) and HPPH2-Cypate (**11**) was also assessed after the photo-induced bleaching experiment, as shown in Figure 4. We observed that there were no PDT cures upon assessment of the tumor response (Figure 4 and Figure 5). All mice were sacrificed when the tumor sizes grew to a volume of 400 mm^3^. The dismal tumor response could be due to changes in tumor oxygenation induced by high fluence rates [31]. High fluence rates such as that used in this experiment (135 J/cm^2^ and 75 mW/cm^2^) can cause the depletion of molecular oxygen during the process of singlet oxygen generation, which can exceed the rate at which it can be resupplied by diffusion from the vasculature [31].

The difference in the rate of in vitro versus in vivo photobleaching could be due to inability of our system to exclude environmental oxygen during the light irradiation and measuring the absorption spectra at various time points, whereas under in vivo conditions, the amount of available oxygen in restricted (it could vary in tumor types). It is believed that such occurrences would lead to PDT self-limiting hypoxic conditions [31] whereby the tumor and surrounding regions become deprived of oxygen. Therefore, we adapted the following experiments with the low-fluence rates. However, for this study, besides **9** (which showed limited in vivo PDT efficacy, Figure 5), we also selected conjugates **6**, **7** and **12**. In our previous study **6** and **7** showed improved long-term cure over 12 at higher light fluence (135 J/cm^2^) and fluence rate (75 mW/cm^2^) [29,30]. The other reason, for the selection of these conjugates was to understand the impact of the length of linkers between the two chromophores as well as the number of HPPH moiety.

### 2.4. In Vivo Photobleaching before and after Low Fluence Light Treatments

**Figure 6:**
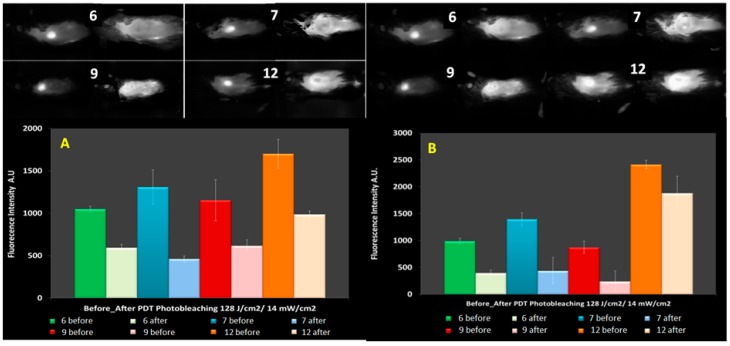
(**A**) Photobleaching of the HPPH portion of PS-CD conjugates and (**B**) a comparative photobleaching of the CD moiety in HPPH-CD conjugates **6**, **7**, **9** and **12** at the light dose of 128 J/cm^2^ and 14 mW/cm^2^.

It has been demonstrated that there was a direct relationship between the tumor response and the level of oxygen within the tumor tissue during PDT [31,32,33,34,35,36,37,38]. Additionally, it has also been demonstrated that using HPPH as a PS and exposing the tumor at low light fluence and fluence rate of 128 J/cm^2^ and 14 mW/cm^2^ showed better tumor response rates with cures up 90 days after PDT [31,33]. Another observation made during these experiments was that the tumor response for the fluence of 128 J/cm^2^ increased as the fluence rate decreased further [31,33]. Therefore, to understand the impact of these light treatment parameters in PDT efficacy of the PS-CD conjugates, the photobleaching experiments were conducted at various fluence and fluence rates of 135 J/cm^2^ and 75 mW/cm^2^; 128 J/cm^2^ and 14 mW/cm^2^ and 128 J/cm^2^ and 7 mW/cm^2^ (Figure 6).

**Figure 7:**
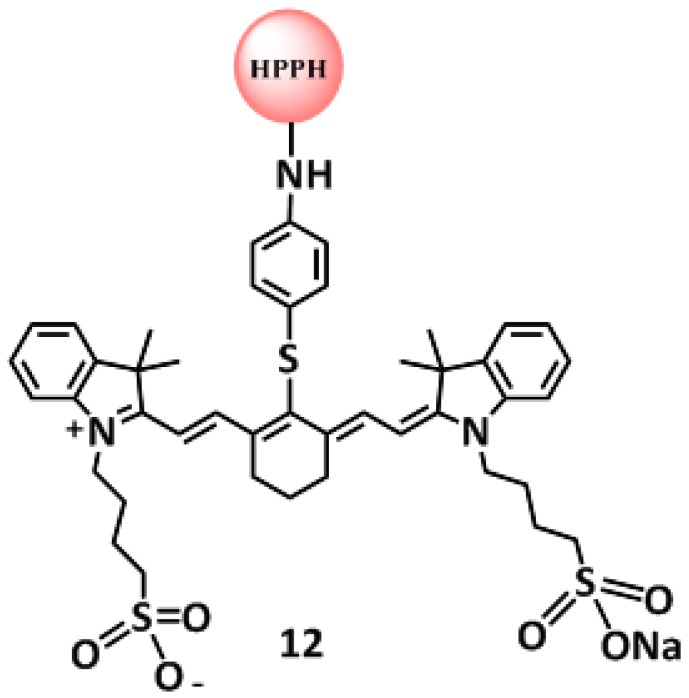
Structure of HPPH-CD conjugate **12**.

In these experiments, the fluorescence was observed before and after PDT treatment at the fluence and fluence rates mentioned above. The compounds chosen for this study were conjugates **6**, **7**, **9** (Figure 2) and **12** (Figure 7) on the basis of their significant in vitro and in vivo PDT responses in mice bearing Colon-26 and U87 tumor models [29,30].The photobleaching data obtained from these experiments were used in conjunction with the molecular modeling of the compounds to infer possible optimization of PDT light dosimetry since it has been reported that it is not simple to predict the photobiological outcome from in vivo photobleaching data alone, because of the complex dependence on oxygenation and micro-environment factors [22]. The fluorescence of HPPH and the CD were also compared prior to light irradiation and at the end of treatment with light dose of 128 J/cm^2^ and 14 mW/cm^2^. After combining the data obtained from the three mice in each group it was found that the HPPH (PS) portion of compounds **6**, **7**, **9** and **12** photobleached by 43%, 65%, 47% and 42% respectively; whereas, the CD portion underwent photobleaching by 60%, 69%, 73% and 22% respectively.

Under similar treatment parameters, the photobleaching rates of the CD portion of the conjugate in **6**, **7** and **9** were observed in the range of 60–73% when irradiated at 128 J/cm^2^ and 14 mW/cm^2^. On the other hand, the PDT response was 40% for conjugate **9** with only 20% photobleaching observed for the CD (Table 1). These results suggest that there is a direct correlation between the rate of photobleaching of the CD and the tumor response; more the photobleaching of the CD in HPPH-CD conjugates, the higher was the tumor response. It was difficult to infer a similar conclusion in the case of HPPH photobleaching with respect to tumor response, since the photobleaching rates were quite similar for all the cases except compound **6**. Summary of photobleaching rates of **6**, **7**, **9** and **12** at 128 J/cm^2^ and 14 mW/cm^2^ are shown in Table 1 and Table 2.

There was no PDT response when a fluence and fluence rate of 128 J/cm^2^ and 7 mW/cm^2^ was used. This can be explained by the threshold dose below which the repair of sub-lethal damage overrides the advantages of low fluence rate [31,39,40]. However, in the experiment where fluence rates of 135 J/cm^2^ and 75 mW/cm^2^ (Table 3) were used, the photo-induced bleaching of the CD portion of the conjugate was between 16% and 52% and there was a tumor response of 66% and 33% for compounds **9** and **12,** respectively. The degree of photobleaching of the HPPH moiety also correlated well with the NIRF moiety. Therefore, the less photobleached the HPPH or NIRF at 135 J/cm^2^ and 75 mW/cm^2^ the better was the response. This could also be due to with the amount accumulated conjugate (PS) within the tumor before PDT light illumination. It has been shown that the combination of fluence and fluence rate can lead to anoxic conditions within the first few minutes of illumination [31,33]. Therefore, it can be deduced that if the amount of photosensitizer accumulated within the tumor prior to PDT is high this would help to circumvent the observance of anoxic conditions.

Experimental:

(a) Chemistry: Compounds investigated in this study were synthesized and characterized by following our own methodology [29,30].
(b) Photophysical characterization: UV-vis absorption spectra were acquired using a Shimadzu UV-3600 spectrophotometer. Fluorescence spectra were recorded using a Fluorolog-3-spectrofluorometer or a SPEX 270M Spectrometer (Jobin Yvon, Longjumeau, France). The SPEX 270 M Spectrometer was utilized for measurement in NIR range; laser lines from Argon ion laser (Spectra Physics) or laser diodes emitting at 630 nm and 785 nm was used as excitation wavelength and the emission was recorded (Table 1, Table 2 and Table 3).
(c) In vitro and in vivo photobleaching: In vitro photobleaching was conducted by observing the UV-vis spectra of conjugates at 5 μM concentrations dissolved either in methanol or in 1% Tween80 formulation diluted with methanol (30-fold, for complete disaggregation of the product). The solution of conjugate in cuvette was then irradiated with light (therapeutic light dose) at various time points for 30 min. At these time intervals, the UV-vis spectrum was taken and the rate of photobleaching (decrease of absorption intensity) at the longest wavelength absorptions of the PS and CD moieties present in PS-CD conjugates were measured and plotted against time.
  To determine the rate of in vivo photobleaching of the PS and CD moieties during the PDT BALB/c mice (5 mice/group) bearing Colon26 tumors were injected (i.v.) with the conjugate(s) and at 24 h post injection (the time point for maximal uptake of the compound). Three sets of therapeutic light doses (light fluence and fluence rates) used for this study were: (135 J/cm^2^, 128 J/cm^2^), 128 J/cm^2^, 14 mW/cm^2^) and (48 J/cm^2^, 7 mW/cm^2^). The fluorescence intensity was measured using the RED (615–665 nm; 750 nm long-pass) and NIR (710–760 nm; 800 nm long pass) excitation and emission before and after PDT for the PS and CD moieties respectively. These experiments were performed using the drug dose of 1.5 μmol /kg for all the conjugates.
(d) In vivo PDT efficacy: Prior to commencement of in vivo studies, all procedures or protocols were approved by the institutional animal care committee (IACUC). In brief, BALB/c mice 5–8 weeks of age were obtained from NCI Jackson Laboratory. The mice were inoculated subcutaneously (S. C.) on the right posterior shoulder with Colon-26 (1 × 10^6^ cells in 50 μL medium) between 7 and 14 weeks of age. The tumors reaching the appropriate treatment size (4–5 mm diameter), the mice were injected with the conjugate intravenously (i.v.) via tail vein injection. At 24 h post injection, the mice were restrained in plexiglass holders without anesthesia, treated with a 1.1 cm diameter area of drug-activating laser light at 665 nm at different light fluence and fluence rates (Table 1 and Table 2). The mice were observed daily for tumor re-growth and tumor cure. Upon tumor recurrence measurements were taken using two orthogonal measurements length and width (perpendicular to L); volumes of tumors were calculated using the Microsoft Excel formula V = L*W^2^/2 and recorded. Mice were considered cured if there was no palpable tumor by day 60; however, if the tumor reached 400 mm in size they were euthanized.
(e) Tumor imaging: BALB/c mice bearing Colon-26 tumors (3 mice/group) were injected (i.v.) with the conjugate (s) and imaged at three time points 24, 48 and 72 h after being anesthetized with Ketamine/Xylazine, delivered intraperitoneally or anesthetized with isoflurane. Compounds were imaged using a Maestro GNIR Flex in vivo imaging system using a broadband excitation at 710–740 nm and 800 nm long pass emission.

## 3. Conclusions

The data acquired from the in vitro photo-induced bleaching of conjugates showed that NIR fluorophore CD of the conjugates photobleached at a much faster rate than HPPH and the rate of bleaching was in the order of **9** > **10** > **8** > **11**, which correlated well with their in vivo PDT responses when irradiated at 128 J/cm^2^ and 14 mW/cm^2^, using a drug dose of 1.5 μmol/kg. Conjugate 12 produced 80% tumor response, whereas compounds 10 and 11 did not yield any long-term cure. The in vivo photobleaching of HPPH and CD in conjugates before and after PDT suggests that measuring the rate of the photobleaching of CD could be a useful tool to optimize the PDT light dosimetry. We hypothesize that the determining the rate of photobleaching of CD in PS-CD conjugates may help to measure indirectly the amount of singlet oxygen generation during photodynamic therapy treatment at variable light fluence and fluence rates [1,22]. These studies are currently underway. A decreased rate of photobleaching of HPPH over the CD in HPPH-CD conjugates could be due to higher reactivity of singlet oxygen to polymethine linkers present in the CD moiety as shown in Figure 1.

According to Wilson et al. [41] the use of photobleaching as a dose metric is based on the fact that the photosensitizer or NIR fluorophore will be degraded directly or indirectly by singlet oxygen as it goes through each photo-activation cycle [41]. An enhanced level of photo-activation is directly proportional to enhanced photobleaching. As a result, a hypothesis is proposed that greater photobleaching represents greater singlet oxygen production and hence an enhanced photodynamic efficacy [41]. However, this assumption/hypothesis may lead to several issues such as: (i) If the fluorescence of the PS is used for the measurements without knowing the absolute initial concentration of the PS, it could be difficult to determine the absolute number of PS molecules photobleached per unit volume during PDT [39]. It has been shown, however, that this requirement can be fulfilled by quantitative fluorescence imaging methods that can quantify absolute PS concentrations (ii) Molecular changes of the PS within tissue may result in changes to the fluorescence without any changes in PDT response [41]; (iii) the photobleaching rate itself may not be a sole indicator for PDT dosimetry/response [41], since it has been shown that singlet oxygen generation is dependent on the tissue microenvironment [4,41]. The micro/local-distribution of the PS will be difficult to quantify because noninvasive or noninvasive fluorescence measurements are limited and can only provide a measure of average/bulk value of the PS concentration.

We agree with the comments of one of the reviewers: “The goal of this study was to ascertain if there was a quantitative relation between photobleaching of the PS and PDT activity. Though that goal was not attained, a semi-quantitative relation between the rate of photobleaching of the CD in HPPH-CD conjugates and various light fluence and fluence rates suggests that this approach with further refinements could be useful for clinicians in identifying the location of the tumors by fluorescence imaging > 830 nm and the depletion of oxygen during the PDT treatment measured by photobleaching of the CD.” These studies with these and other PS-CD conjugates are currently in progress.

## Figures and Tables

**Table 1:** Summary of a comparative in vivo photobleaching of HPPH moiety in HPPH-CD conjugates **6**, **7**, **9** and **12** after the light treatment (128 J/cm^2^ and 14 mW/cm^2^) for 30 min. The tumors were exposed to light at 665 nm (in vivo absorption of HPPH at its longest wavelength absorption) after 24 h post-injection of the conjugates.

Compounds		Fluorescence (Not an Absolute Value)				Photobleached 128 J/cm^2^ 14 mW/cm^2^		% Tumor Cured 128 J/cm^2^ 14 mW/cm^2^
Drug#	Dose (μmol/kg)	Pre PDT HPPH	Pre PDT CD	Post PDT HPPH	Post PDT CD	% HPPH Photo-Bleached	% CD Photo-Bleached	PDT ONLY
**6**	1.5	1048	989	592	397	43	60	80
**7**	1.5	1308	1396	460	439	65	69	80
**9**	1.5	1154	874	616	239	47	73	80
**12**	1.5	1701	2413	986	1881	42	22	40

**Table 2:** Summary of the photobleaching results with conjugates **6**, **7**, **9** and **12** at a light dose of 48 J/cm^2^ and 7 mW/cm^2^. The PDT activity of the conjugates at a light dose of 48J/cm^2^, 7 mW/cm^2^ was not determined (ND).

Compounds		Fluorescence (Not an Absolute Value)				Photobleached 48 J/cm^2^ 7 mW/cm^2^		% Tumor Cured 48 J/cm^2^ 7 mW/cm^2^
Drug#	Dose (μmol/kg)	Pre PDT HPPH	Pre PDT CD	Post PDT HPPH	Post PDT CD	% HPPH Photo-Bleached	% CD Photo-Bleached	PDT ONLY
**6**	1.5	1060	976	825	492	22	50	ND
**7**	1.5	1485	1319	1170	1019	21	23	ND
**9**	1.5	986	809	808	459	18	43	ND
**12**	1.5	1428	2538	1238	1783	13	30	ND

**Table 3:** Summary of the photobleaching of conjugates **6**, **7**, **9** and **12** at 135 J/cm^2^ and 75 mW/cm^2^.

Compounds		Fluorescence (Not an Absolute Value)				Photobleached 135 J/cm^2^ 75 mW/cm^2^		% Tumor Cured 135 J/cm^2^ 75 mW/cm^2^
Drug#	Dose (μmol/kg)	Pre PDT HPPH	Pre PDT CD	Post PDT HPPH	Post PDT CD	% HPPH Photo-Bleached	% CD Photo-Bleached	% Tumor Cure
**6**	1.5	1136	1025	670	416	41	60	0
**7**	1.5	1380	1319	655	560	53	58	0
**9**	1.5	996	904	683	430	31	52	33
**12**	1.5	1392	2543	1390	2143	8	16	66
